# Kudzu Leaf Extract Suppresses the Production of Inducible Nitric Oxide Synthase, Cyclooxygenase-2, Tumor Necrosis Factor-Alpha, and Interleukin-6 via Inhibition of JNK, TBK1 and STAT1 in Inflammatory Macrophages

**DOI:** 10.3390/ijms19051536

**Published:** 2018-05-22

**Authors:** Seok Hyun Eom, So-Jung Jin, Hee-Yeong Jeong, Youngju Song, You Jin Lim, Jong-In Kim, Youn-Hyung Lee, Hee Kang

**Affiliations:** 1 Department of Horticultural Biotechnology, College of Life Sciences, Kyung Hee University, Yongin 17104, Korea; se43@khu.ac.kr (S.-H.E.); truesojung@gmail.com (S.-J.J.); yujn0213@naver.com (Y.-J.L.); younlee@khu.ac.kr (Y.-H.L.); 2 Graduate School of East-West Medical Science, Kyung Hee University, Yongin 17104, Korea; jhy9592@naver.com; 3 Department of Biomedical Science and Technology, Graduate School, Kyung Hee University, Seoul 02447, Korea; songyj@khu.ac.kr; 4 Division of Acupuncture and Moxibustion Medicine, Kyung Hee Korean Medicine Hospital, Kyung Hee University, Seoul 02447, Korea; hann8400@hanmail.net

**Keywords:** kudzu leaf, robinin, inflammation, macrophages, inducible nitric oxide synthase, STAT1

## Abstract

Kudzu (*Pueraria montana* var. *lobata* (Willd.) Sanjappa & Pradeep) is a perennial leguminous vine, and its root and flower have been used for herbal medicine in Asia for a long time. Most dietary flavonoids are reported to be concentrated in its root, not in its aerial parts including leaves. In this study, we investigated whether kudzu leaf and its major constituent, robinin (kaempferol-3-*O*-robinoside-7-*O*-rhanmoside) possessed anti-inflammatory activity. To test this hypothesis, we used peritoneal macrophages isolated from BALB/c mice and stimulated the cells with lipopolysaccharide (LPS) or LPS plus interferon (IFN)-γ. Compared with kudzu root extract, its leaf extract was more potent in inhibiting the production of inducible nitric oxide synthase (iNOS), cyclooxygenase-2, tumor necrosis factor-α, and interleukin-6. Kudzu leaf extract decreased LPS-induced activation of c-Jun N-terminal kinase (JNK) and TANK-binding kinase 1(TBK1) with no effects on nuclear factor-κB and activator protein 1 transcriptional activity. Also, kudzu leaf extract inhibited LPS/IFN-γ-induced signal transducer and activator of transcription 1 (STAT1) activation partly via an altered level of STAT1 expression. Robinin, being present in 0.46% of dry weight of leaf extract, but almost undetected in the root, decreased iNOS protein involving modulation of JNK and STAT1 activation. However, robinin showed no impact on other inflammatory markers. Our data provide evidence that kudzu leaf is an excellent food source of as yet unknown anti-inflammatory constituents.

## 1. Introduction

Inflammation is a protective response that eliminates noxious stimuli and promotes the regeneration of damaged tissue. Generally, this response begins when phagocytes detect harmful substances from microbes or dying cells through pattern recognition receptors such as toll-like receptors (TLRs) [1]. Several peptide and lipid mediators are synthesized by phagocytes and other immune cells nearby, resulting in vasodilation, increased capillary permeability, and vascular stickiness [1]. This brings white blood cells out of the blood vessels to migrate to the site and participate in the ongoing inflammatory response in cooperation with the resident phagocytes. Because excessive production of inflammatory mediators can be deleterious to normal tissue, the whole process should be tightly controlled.

Macrophages, one type of the professional phagocytes, play a critical role in inflammation. Macrophages generate inflammatory response to lipopolysaccharide (LPS) through the TLR4/MD-2 complex. The cytoplasmic tail of TLR4 uses two sets of adaptor proteins: MAL-MyD88 at the plasma membrane and TRAM-TRIF at the endosomal membrane [2]. The MAL-MyD88 pathway triggers the initial activation of nuclear factor-κB (NF-κB) and mitogen-activated protein kinase (MAPK)s, resulting in the production of inflammatory proteins such as inducible nitric oxide synthase (iNOS), cyclooxygenase (COX)-2, tumor necrosis factor (TNF)-α, and interleukin (IL)-6 [3,4]. NF-κB activation occurs when IκBα, which keeps NF-κB in the cytosol under resting conditions, is degraded, allowing free NF-κB to migrate to the nucleus and bind to κB sites in various inflammatory gene promoters [5]. MAPKs (p38, c-Jun N-terminal kinase (JNK), and extracellular signal-regulated kinase (ERK)1/2) regulate the activity of activator protein 1 (AP-1) and other transcription factors necessary for inflammation or directly regulate the mRNA stability of inflammatory genes [6,7]. The TRAM-TRIF pathway leads to the activation of interferon regulatory factor (IRF)-3 and the transcription of interferon (IFN)-β and the late-phase activation of NF-κB and MAPKs [2]. Signal transducer and activator of transcription 1 (STAT1) is a latent cytosolic transcription factor that mediates INF-γ-dependent gene expression including iNOS [8,9,10]. IFN-γ, originally called ‘macrophage activating factor’, primes macrophages for enhanced response to TLR and amplifies TLR-induced NF-κB activation [11]. When IFN-γ binds to its receptor, the receptor-associated janus kinase (JAK)1 and JAK2 phosphorylate STAT1, leading to the dimerization of the protein, its migration to the nucleus, and binding to the DNA in target gene promoters [12].

Kudzu (*Pueraria montana* var. *lobata* (Willd.) Sanjappa & Pradeep) is a perennial and leguminous vine native to Southeast Asia and was introduced to North America in the late 1800s to feed livestock and prevent soil erosion. Kudzu is resistant to insect pests and drought and is one of the energy crops in the US [13]. On the other hand, the growth of kudzu is so aggressive that it destroys native vegetation, giving it the status of a pest species [14]. Kudzu root has a long history of medicinal use for fever, diarrhea, diabetes, and hangover in China, Japan and Korea [15]. Not only the root but also the flower has been used for alcohol intoxication [15]. Most biological studies have been focused on the kudzu root and flower. In addition, kudzu leaves are edible and used in various foods, as are the root and flower. Kudzu leaves contain kakkalide, genistin, rutin, robinin (kaempferol-3-O-robinoside-7-O-rhamnoside), nicotiflorin (kaepmferol-3-O-rutinoside), and kaikosaponin III [16]. In this study, we investigated whether the kudzu leaf extract showed any inflammatory effects on the production of iNOS, COX-2, TNF-α, and IL-6 in macrophages and compared its efficacy with that of the kudzu root extract. Further, we tried to establish the underlying mechanism of kudzu leaf extract during stimulation with LPS or LPS plus IFN-γ. We also characterized the activity of robinin, a major constituent of kudzu leaf extract.

## 2. Results

### 2.1. Effects of Kudzu Leaf Extract on Cell Viability and the Production of Inducible Nitric Oxide Synthase (iNOS) and Nitric Oxide in Mouse Peritoneal Macrophages

**Figure 1:**
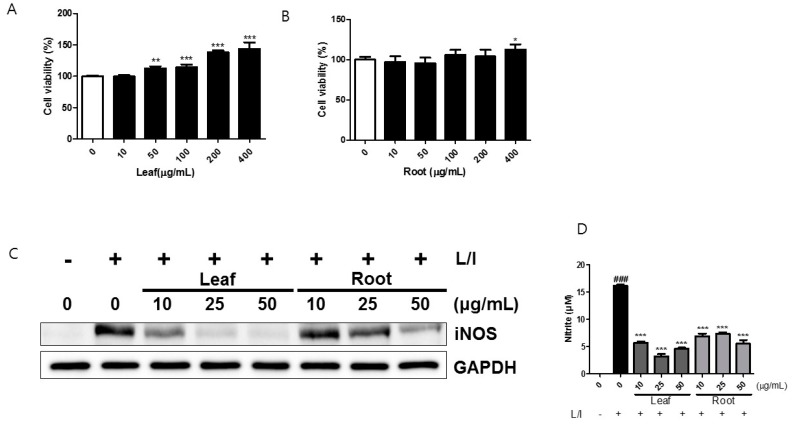
Effects of kudzu leaf and root extracts on cell viability and the production of inducible nitric oxide synthase (iNOS) and nitric oxide (NO). (**A**,**B**): Mouse peritoneal macrophages isolated from BALB/c mice were cultured with kudzu (**A**) leaf extract or (**B**) root extract for 24 h. Cell viability was determined using the MTT assay. Data are represented as percentages of control cells (0 μg/mL extract) (*n* = 4). * *p* < 0.05, ** *p* < 0.01, *** *p* < 0.005 vs. control. (**C**,**D**): Mouse peritoneal macrophages were stimulated with LPS (L) and IFN-γ (I) in the presence of kudzu leaf or root extract for 24 h. Whole cell protein was extracted and the level of iNOS protein was analyzed by Western blotting using GAPDH as an internal control. One of five independent experiments is shown. The nitrite accumulation in the supernatant was measured by the Griess reagent assay (*n* = 3). ### *p* < 0.005 vs. control (−L); *** *p* < 0.005 vs. control (+L).

First, we measured the effects of kudzu leaf and root extracts on cell viability using the MTT method. Mouse peritoneal macrophages were treated with increasing concentrations of leaf or root extract. Concentrations of both types of extracts up to 400 μg/mL were not cytotoxic to peritoneal macrophages (Figure 1A,B). We first examined whether kudzu leaf extract affects LPS-induced iNOS production in peritoneal macrophages. Because we isolated peritoneal macrophages from BALB/c mice, a Th2-dominant strain, these cells require IFN-γ to express LPS-induced iNOS and nitric oxide (NO) production [17]. Our preliminary tests showed that kudzu leaf extract completely inhibited iNOS production at 100 μg/mL. Thus, we limited the maximum concentration to 50 μg/mL and compared the potency of the leaf extract with that of the root extract. Decreases in the iNOS protein band were observed in cells treated with a concentration as low as 10 μg/mL of leaf extract. The root extract also inhibited iNOS protein in a dose-dependent manner, but the inhibitory activity of the leaf extract was much stronger than that of the root extract (Figure 1C). Subsequently NO generation in supernatant was measured using the Griess reaction. Nitrite accumulation was used as an indicator of NO generation. Likewise, the leaf extract was more potent than the root extract in decreasing nitrite accumulation (Figure 1D).

### 2.2. Effects of Kudzu Leaf Extract on Cyclooxygeanse-2, Tumor Necrosis Factor-α, and Interleukin-6 in Mouse Peritoneal Macrophages

**Figure 2:**
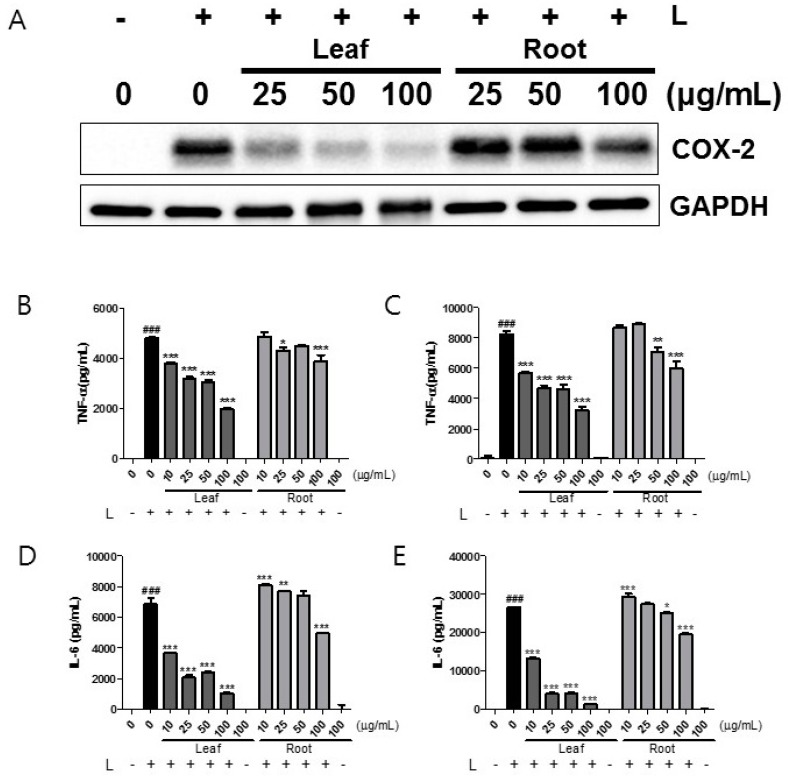
Effects of kudzu leaf and root extracts on cyclooxygenase (COX)-2, tumor necrosis factor (TNF)-α, and interleukin (IL)-6 in mouse peritoneal macrophages. (**A**): Mouse peritoneal macrophages were stimulated with LPS (L) in the presence of kudzu leaf or root extract for 24 h. The level of COX-2 protein was analyzed by Western blotting using GAPDH as an internal control. One of three independent experiments is shown. (**B**–**E**): Mouse peritoneal macrophages were stimulated with LPS (L) for 24 h, and levels of TNF-α and IL-6 at 6 h (**B**,**D**) and 24 h (**C**,**E**) were measured by enzyme-linked immunosorbent assay (ELISA) (*n* = 3). ### *p* < 0.005 vs. control (−L); * *p* < 0.05, ** *p* < 0.01, *** *p* < 0.005 vs. control (+L).

Next, we measured the effects of kudzu leaf extract on COX-2 production in LPS-stimulated macrophages. Cells were stimulated with LPS in the presence of leaf or root extract at 25, 50, and 100 μg/mL. The leaf extract was much more potent than the root extract in inhibiting COX-2 production (Figure 2A). A concentration as low as 25 μg/mL of leaf extract clearly suppressed COX-2 while higher concentrations (above 100 μg/mL) of root extract were required to decrease it. Finally, we examined whether kudzu leaf extract influences LPS-stimulated TNF-α and IL-6 production. At 6 h (Figure 2B,D) and 24 h (Figure 2C,E) time points, the leaf extract decreased the levels of TNF-α and IL-6 more potently than did the root extract. Cells treated with leaf or root extract alone did not produce any detectable levels of each cytokine.

### 2.3. Effects of Kudzu Leaf Extract on Nuclear Factor-κB, Mitogen-Activated Protein Kinase, and TABK-Binding Kinase 1 Activation in Mouse Peritoneal Macrophages

**Figure 3:**
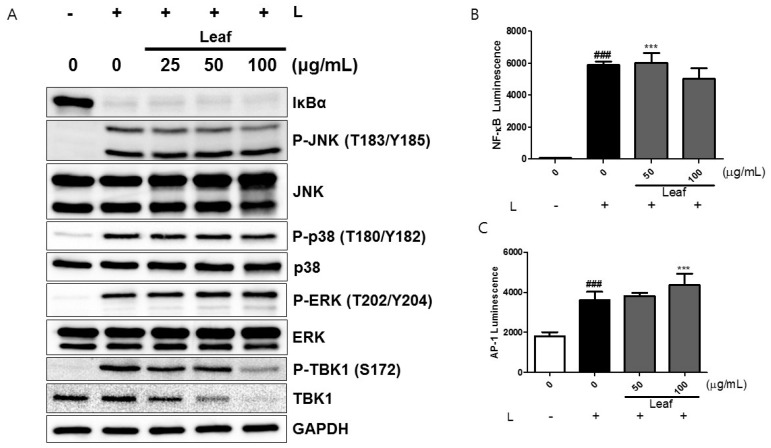
Effects of kudzu leaf extract on nuclear factor-κB (NF-κB), mitogen-activated protein kinase (MAPK), activator protein 1 (AP-1), and Tank-binding kinase 1 (TBK1) activation. (**A**) Mouse peritoneal macrophages were treated with kudzu leaf extract for 1 h followed by stimulation with LPS (L) for 15 min. Whole cell extracts were prepared and subjected to Western blotting. The level of signaling proteins was analyzed using GAPDH as an internal control. One of three independent experiments is shown. (**B**,**C**) RAW264.7 cells were transfected with an NF-κB-or AP-1-dependent reporter gene. Cells were pretreated with kudzu leaf extract for 2 h and then stimulated with LPS for 6 h. Luciferase activity was measured using the Dual-Glo^®^ luciferase assay system (*n* = 3). ### *p* < 0.005 vs. control (−L); *** *p* < 0.005 vs. control (+L).

We investigated any effect of kudzu leaf extract on the degradation of IκBα, a critical step in NF-κB and signaling [18]. Cells stimulated with LPS for 15 min showed complete degradation of IκBα and kudzu leaf extract did not influence this process (Figure 3A). We also examined whether kudzu leaf extract affects NF-κB transcriptional activity using luciferase reporter assays. Kudzu leaf extract alone did not stimulate NF-κB activity (Figure S1). LPS increased NF-κB transcriptional activity and kudzu leaf extract did not show any inhibitory effects (Figure 3B). We examined MAPK activation and AP-1 transcriptional activity. Stimulation with LPS for 15 min caused the phosphorylation of JNK, p38, and ERK1/2 (Figure 3A). Among these MAPKs, kudzu leaf extract decreased the expression of phosphorylated JNK but rather enhanced the transcriptional activity of AP-1 (Figure 3C). We investigated whether kudzu leaf extract may interfere with TANK-binding kinase 1 (TBK1) phosphorylation as a measure of TRIF signaling. Kudzu leaf extract decreased TBK1 phosphorylation (Figure 3A). Interestingly, it also decreased TBK1 protein expression in a dose-dependent manner, indicating that the decrease in TBK1 phosphorylation by kudzu leaf extract was mediated by its decrease in TBK1 protein itself.

### 2.4. Effects of Kudzu Leaf Extract on Signal Transducer and Activator of Transcription 1 Activation in Mouse Peritoneal Macrophages

**Figure 4:**
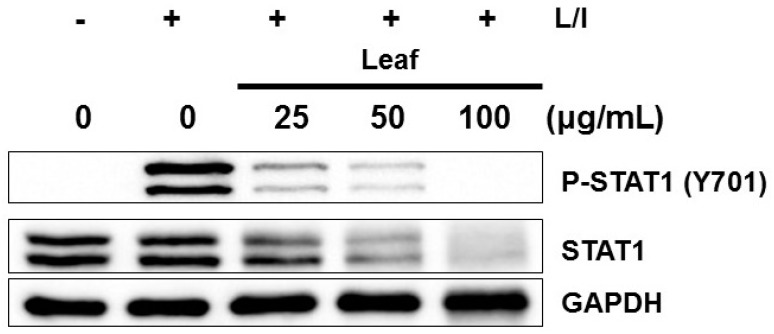
Effect of kudzu leaf extract on signal transducer and activator of transcription 1 (STAT1) activation. Mouse peritoneal macrophages were treated with kudzu leaf extract for 1 h followed by stimulation with LPS (L) and IFN-γ (I) for 2 h. Whole cell extracts were prepared and subjected to Western blotting. The level of signaling proteins was analyzed using GAPDH as an internal control. One of three independent experiments is shown.

Because the strong inhibition of iNOS production by kudzu leaf extract did not appear to be related to NF-κB and AP-1, we investigated whether this reduction may be associated with the STAT1 pathway that is required for iNOS expression [9,10]. Cells were stimulated with LPS and IFN-γ to maximize STAT1 activation [8]. We measured phosphorylation of STAT1 on tyrosine 701. Kudzu leaf extract strongly inhibited STAT1 phosphorylation in a dose-dependent manner: STAT1 activation was abrogated at 100 μg/mL kudzu leaf extract (Figure 4). Notably, decreases in STAT1 were observed at increasing concentrations of kudzu leaf extract. 

### 2.5. High-performance Liquid Chromatography (HPLC) Analysis of Robinin and Puerarin in Kudzu Leaves and Roots

**Figure 5:**
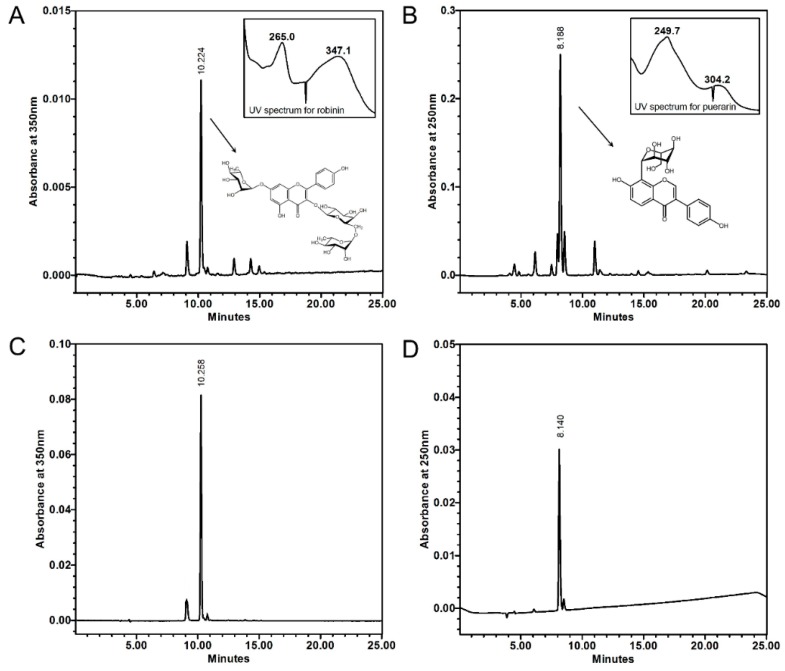
HPLC chromatograms of kudzu leaf extract (**A**), root extract (**B**), robinin (**C**), and puerarin (**D**).

A comparison of the contents of genistein, genistin, daidzein, daidzin, and puerarin between kudzu leaf and root was reported [19]: the leaf contained 1/10 to 1/40 of these isoflavonoids compared with the root. Here, we chose robinin and puerarin, the known major isoflavone in kudzu root, and compared their contents. In the high-performance liquid chromatography (HPLC) analysis of leaf extracts, one major substance was detected at a retention time of 10.2 min on the 350 nm ultraviolet (UV)-spectrum (Figure 5A). The compound was confirmed as robinin by photodiode array spectral analysis of HPLC using λ_max_ = 265.0 and 347.1 nm values and also by ^1^H- and ^13^C-NMRs (Figure S2) after the isolation process of the compound. Robinin showed a purity of 90%, based on the peak area at 350 nm detection of HPLC chromatogram (Figure 5C). Puerarin in the root extract was detected at a retention time of 8.2 min on the 250 nm UV-spectrum, exhibiting λ_max_ = 249.7 and 304.2 nm (Figure 5B). The compound was confirmed by HPLC analysis of standard puerarin (Figure 5D). The yields of the methanol extracts from the leaves and roots were about 11% and 25%, respectively (Table 1). Robinin was present in about 0.46% of the dry weight of leaf extract and was not detected in the roots (Figure 3, Table 1). In contrast, puerarin was abundantly present in about 4.35% of the root dry weight and was not detected in the leaf extract. This pattern was similar to the content of *Pueraria montana* reported by Kirakosyan et al. [19], in which the puerarin level in the roots showed the highest value, while far lower amounts of puerarin were found in the leaves and other organs.

### 2.6. Effects of Robinin on iNOS Production and Signaling Molecules in Activated Macrophages

**Figure 6:**
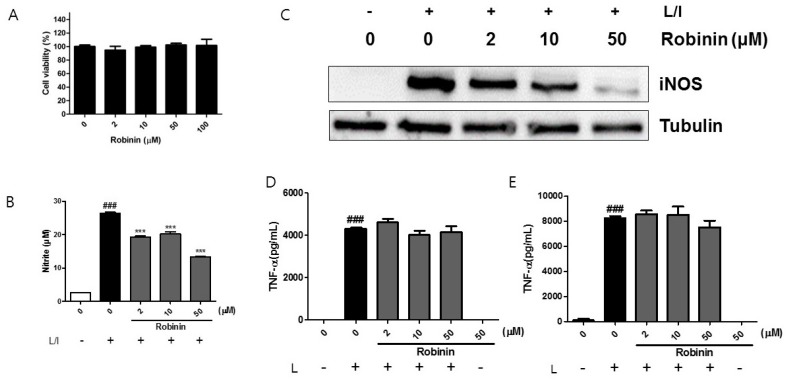
Effect of robinin on inflammatory responses in mouse peritoneal macrophages. (**A**) Mouse peritoneal macrophages were cultured with robinin for 24 h. Cell viability was determined using the MTT assay. Data are represented as percentage of control cells (0 μg/mL robinin) (*n* = 4). (**B**,**C**) Cells were stimulated with LPS (L) and IFN-γ (I) in the presence of robinin for 24 h. The level of iNOS protein (**B**) was analyzed by Western blotting using tubulin as an internal control. One of three independent experiments is shown. The levels of nitrite (**C**) were measured using the Griess reaction. (**D**,**E**) The levels of TNF-α at 6 h (**D**) and 24 h (**E**) in the supernatant was measured by ELISA (*n* = 3). ### *p* <0.005 vs. control (−L); *** *p* < 0.005 vs. control (+L/I).

**Figure 7:**
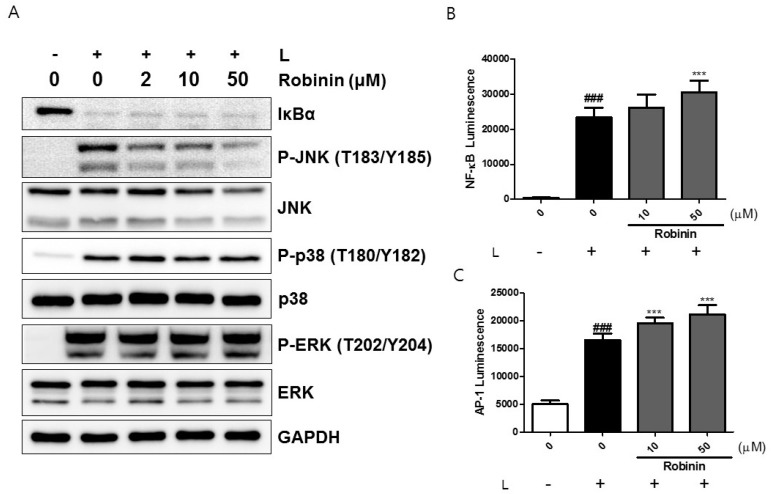
Effects of robinin on LPS-induced NF-κB and MAPK activation in mouse peritoneal macrophages. (**A**) Mouse peritoneal macrophages were treated with robinin for 1 h followed by stimulation with LPS (L) for 15 min. Whole cell extracts were prepared and subjected to Western blotting. The level of signaling proteins was analyzed using GAPDH as an internal control. One of three independent experiments is shown. (**B**,**C**): RAW264.7 cells were transfected with an NF-κB (**B**) or AP-1 (**C**)-dependent reporter gene. Cells were pretreated with robinin for 2 h and then stimulated with LPS for 6 h. Luciferase activity was measured using the Dual-Glo^®^ luciferase assay system (*n* = 3). ### *p* <0.005 vs. control (−L); *** *p* < 0.005 vs. control (+L).

**Figure 8:**
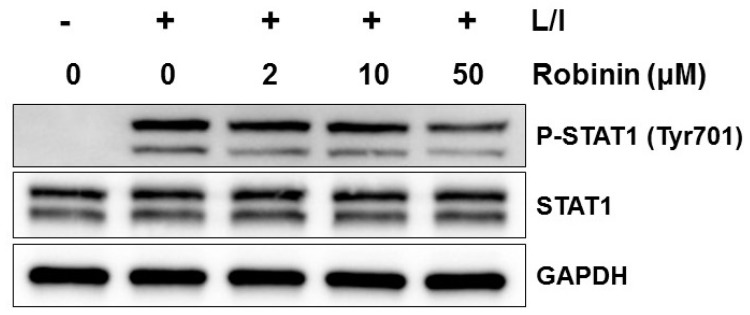
Effect of robinin on LPS/IFN-γ-induced STAT1 activation in mouse peritoneal macrophages. Mouse peritoneal macrophages were treated with robinin for 1 h followed by stimulation with LPS (L) and IFN-γ (I) for 2 h. Whole cell extracts were prepared and subjected to Western blotting. The level of signaling proteins was analyzed using GAPDH as an internal control. One of three independent experiments is shown.

Because robinin exists abundantly in kudzu leaf, we examined whether this compound might affect the iNOS protein levels in activated macrophages. We confirmed that robinin concentrations up to 100 μM did not show any cytotoxicity (Figure 6A). Robinin decreased LPS/IFN-γ-induced nitrite and iNOS production in a dose-dependent manner (Figure 6B,C). However, LPS-induced production of TNF-α at 6 and 24 h was unaltered (Figure 6D,E). No change in COX-2 and IL-6 was observed (Figure S3). Robinin itself did not stimulate NF-κB transcriptional activity (Figure S1) but rather enhanced LPS-induced NF-κB transcriptional activity without effects on IκBα degradation (Figure 7A,B). Robinin inhibited JNK activation but enhanced the transcriptional activity AP-1 (Figure 7A,C). A robinin concentration of 50 μM inhibited STAT1 phosphorylation (Figure 8).

## 3. Discussion

In comparison to kudzu root and flower, which have a long history of medical use, little attention has been paid to kudzu leaf. In this sense, our study shed light on the importance of kudzu leaf as a rich source of anti-inflammatory constituents. Kudzu leaf extract was much more potent than its root extract in inhibiting the important inflammatory markers such as iNOS, COX-2, TNF-α, and IL-6 in macrophages stimulated with LPS or LPS plus IFN-γ.

In macrophages, iNOS catalyzes the conversion of L-arginine, molecular oxygen, and NADPH to l-citrullin, water, and NO [20]. NO reacts with the thiol groups of cysteins and forms *S*-nitrosothiols, altering protein function and reacting with superoxide to become the more toxic peroxynitrite, thus causing oxidation and DNA damage [20]. COX-2 catalyzes the conversion of arachidonic acid to prostaglandin H_2_, which leads to the formation of various prostanoids [21]. Prostagladin E_2_, one type of prostanoids, elicits vasodilation, vascular hyperpermeability, fever, and pain. TNF-α and IL-6 activate endothelial cells and other leukocytes and induce the acute-phase response [1]. Therefore, these inflammatory proteins are molecular targets for the development of anti-inflammatory agents.

Many inflammatory genes such as TNF-α, IL-6, COX-2, and iNOS are co-dependent on MyD88 and TRIF but the precise role of the two pathways in expressing these genes seems to be distinct [4,22]. For example, LPS-induced TNF-α production is impaired in MyD88-or TRIF-deficient macrophages [22,23]. However, the MyD88 pathway upregulates TNF-α gene via activation of NF-κB while the TRIF pathway regulates TNF-α promoter activity with little dependence on NF-κB [24]. Several studies indicate that the contribution of the TRAM-TRIF pathway to NF-κB signaling is minor and delayed relative to the MyD88 pathway [3,24,25]. Our data showed that the MyD88-dependnet NF-κB and AP-1 transcriptional activity was the least likely target of kudzu leaf extract. Instead, the suppressive effect of kudzu leaf extract on TBK1 phosphorylation, upstream of IRF-3 activation, may account for one possible mechanism involving the TRIF pathway that mediates inflammatory response independently of NF-κB and AP-1. Many inflammatory genes including TNF-α, IL-6, and COX-2 contain AU-rich elements (ARE) in the 3′-untranslated regions, which determines the rate of mRNA stability and translation [26]. This sequence is regulated by various ARE-binding proteins. In addition to the modulation of AP-1 and NF-κB, MAPKs participate in the inflammatory response by regulating the stability of cytokine genes at the post-transcriptional level. JNK is required for LPS-induced TNF-α and iNOS mRNA translation [27,28]. It is possible that without the involvement of AP-1 transcriptional activity, kudzu leaf extract may affect thee inflammatory gene mRNA stability via modulation of JNK activation.

Among the inflammatory proteins tested here, iNOS responded more sensitively to kudzu leaf extract than the other inflammatory markers. Moreover, the suppressive effect of kudzu leaf extract on STAT1 activation was remarkable. Because STAT1 is required for iNOS expression in macrophages, the inhibition of iNOS and STAT1 by kudzu leaf extract might be correlated. In addition to its direct involvement of iNOS expression, STAT1 contributes to other TLR-induced inflammatory responses: peritoneal macrophages from STAT1 deficient mice showed lower levels of LPS-induced TNF-α and IL-6 production compared to those from wild type mice [29]. This is due to the fact that crosstalk between IFNs and TLR signaling pathways occurs at multiple levels [12,30]. In response to LPS, macrophages activate themselves in an autocrine and paracrine manner by producing IFN-α/β and, to a lesser degree, IFN-γ [31]. IFN-α/β and IFN-γ share the JAK/STAT1 pathway [8]. Thus, inhibition of STAT1 by kudzu leaf extract may confer its anti-inflammatory effects by giving negative feedback to LPS-induced signaling. An interesting observation is that kudzu leaf extract decreases the level of STAT1 protein in a dose-dependent manner. Therefore, the observed inhibitory effect of kudzu leaf extract on STAT1 activation was partially due to an altered level of STAT1 protein. We do not know the mechanism by which kudzu leaf extract causes the degradation of STAT1. Macrophages from IFN-β(−/−) mice showed less STAT1 protein than macrophages from wild type mice [32]. Whether the decrease in STAT1 by kudzu leaf extract is associated with IFN-β signaling remains to be determined.

An analysis of the quantities of isoflavonoids in each organ of kudzu showed that the root contains much higher levels of medicinally important isoflavonoids such as daidzein, daidzin, genistein, genistin, and puerarin than do its aerial parts including leaves [19]. Daidzein, genistein, and puerarin are reported to decrease LPS-induced iNOS production in macrophages [33,34]. A comparative study on the effects of various flavonoids demonstrated that genistein decreased TNF-α production in LPS/IFN-γ-activated macrophages while daidzein, daidzin, and genistin increased its production in a dose-dependent manner [35]. Although kudzu leaves are not reported to be a good source of the known beneficial flavonoids, they seem to contain unidentified bioactive components that exert anti-inflammatory activity. Given the amount of robinin contained in the effective dose of kudzu leaf extract (100 μg/mL of kudzu leaf extract contains about 0.46 μg/mL or 0.6 μM) and its lack of some biological activities such as TNF-α reduction, the anti-inflammatory effect of kudzu leaf is clearly not dependent on robinin.

Robinin is found in other plants such as some species from the *Robinia* and *Astragalus* genera and *Vinca erecta* [36,37]. Robinin is one of the major active components of Flaronin, which is a pharmaceutical product prepared from the leaves and flowers of *Astragalus falcatus* L. in Russia and Georgia and has been shown to enhance the nitrogen-excretory function of kidneys and increase diuresis [37]. Kaempferol, the parent aglycone of robinin, is one of the most common dietary flavonoids and inhibits the expression of iNOS, COX-2, and TNF-α through its modulation of NF-κB, AP-1, and STAT1 in LPS-stimulated macrophages [33,38]. Generally, the potency of glycosides has been demonstrated to be weaker than that of their aglycones because they are less penetrable to cell membranes. We found that kaempferol was much more potent in inhibiting iNOS production than was robinin (data not shown). The downregulation of iNOS by robinin seemed to be mediated by its inhibition of JNK and STAT1. Robinin can be hydrolyzed to kaempferol by intestinal microflora [39]. This suggests that robinin might exert a more potent inhibitory effect when it is consumed orally and converted to kaempferol in the gut.

Taken together, we discovered that despite being low in content of the known anti-inflammatory isoflavonoids, kudzu leaves showed more potent anti-inflammatory activity in macrophages than did kudzu roots. Kudzu leaf extract suppressed LPS-induced production of iNOS, COX-2, TNF-α, and IL-6 via modulation of JNK, TBK1, and STAT1 activation. We also identified that robinin, a major component of kudzu leaf, suppressed iNOS production through the modulation of JNK and STAT1 activity. However, robinin did not represent the anti-inflammatory activity of kudzu leaf extract. Our data provide evidence that in addition to the use of kudzu root as a medicinal and food resource, kudzu leaf is an excellent source of as yet unknown anti-inflammatory constituents.

## 4. Materials and Methods

### 4.1. Sample Preparation

Leaves and roots of kudzu were harvested at the boundary hill between an agricultural field and mountain in Yongin, Korea in July 2014. The fully expanded young leaves were chosen, and the tap roots were selected by removal of the fine roots. After being rinsed three times with distilled water, the leaves and sliced roots (3 mm in thickness) were dried in a 38 °C dry oven for five days and ground with a commercial home grinder. The samples were stored at −20 °C until extraction.

### 4.2. Extraction Procedure

Leaf or root power (each 750 g) was divided equally into three groups. Each sample (250 g) was suspended in 5 L of methanol and kept in a 4 °C cold chamber for 48 h for extraction. Experiments were carried out in triplicate. The suspension was collected after a filtering process, and the debris was suspended again in 5 L of methanol. These steps were repeated until the solvent color disappeared completely to the naked eye. After pooling the suspensions, a vacuum reflux cooling system in a rotary evaporator (N-1100, Eyelar Co., Tokyo, Japan) was applied to evaporate the solvent from the extracts. The samples were then freeze-dried. For cell culture, these extracts were dissolved in dimethyl sulfoxide (DMSO).

### 4.3. Isolation of Robinin

The solvent fractionations using the principle of solvent polarity were performed with *n*-hexane and ethylacetate. Leaf extracts (30 g) attained by the procedure of methanol extraction were dissolved into 1 L of distilled water set into a fraction funnel (3 L volume size) and 1 L of *n*-hexane. The complex solvents were shaken strongly and kept silently for 30 min. After stabilization of the fraction reaction, the *n*-hexane fraction was removed from the water-dissolved extract. After repeating the procedure of *n*-hexane fractionation, further fractionation using ethylacetate with the same procedure as that for *n*-hexane fractionation was performed. A compound that was crystallized during the ethylacetate fractionation in water-dissolved leaf extract was separated by filtering and several washing processes.

### 4.4. HPLC Analysis

Puerarin, the isolated compound, and each extract of leaf and root were dissolved in methanol to a concentration of 0.5 mg/mL, and filtered with a syringe filter (0.45 µm, Futecs, Daejeon, Korea). The HPLC procedure used here was performed similarly to that described by Seo et al. [40], except for the running combination of the mobile phases, solvent A (0.1% formic acidic water) and solvent B (0.1% formic acidic acetonitrile). The mobile phases ran at 15% solvent B for 0–5 min, 20% solvent B for 5–15, 30% solvent B for 15–20 min, and 35% solvent B for 20–23 min. The HPLC system was a Waters 2695 separation module (Waters, Milford, MA, USA) with a Waters 996 photodiode array detector set to 350 nm for robinin and 250 nm for puerarin. The column was a 250 × 4.6 mm Prontosil HPLC column (120-5-C18-ace-EPS 0.5 μm, Bischoff, Snackenberg, Germany). The sample injection volume was 10 μL. The flow rate was 0.8 mL/min, and the column temperature was kept at 30 °C.

### 4.5. Isolation of Mouse Peritoneal Macrophages

Seven-week-old male BALB/c mice were obtained from DBL (Eumsung, Korea) and housed in a temperature- and humidity-controlled pathogen-free animal facility with a 12-h light-dark cycle. The animal protocol (KHUASP(SE)-15-012) was approved by our institutional committee, and mice were cared for according to the US National Research Council for the Care and Use of Laboratory Animals specifications (1996). Mice were injected intraperitoneally with 2 mL of 3.5% sterile thioglycollate solution (BD, Sparks, MD, USA), and four days later, mice were sacrificed by cervical dislocation. Peritoneal exudate cells were isolated by peritoneal lavage with cold DMEM (Hyclone, Logan, UT, USA). After centrifugation, cells were resuspended in DMEM with 10% fetal bovine serum (FBS) (Hyclone, Logan, UT, USA) and 1% penicillin-streptomycin. Cells were plated overnight at 37 °C, and non-adherent cells were removed.

### 4.6. Cell Viability Assay

Cells were seeded in quadruplicate in 96-well plates overnight and the culture medium was removed. Wells were replenished with new medium containing experimental samples for 24 h, and then cell viability was determined using the MTT assay. Briefly, the culture medium was removed and the cells were washed with phosphate buffered saline (PBS). Then, MTT (Sigma, St. Louis, MO, USA) dissolved in DMEM was added to each well to attain a final concentration of 0.5 mg/mL. After 1 h of incubation at 37 °C, the media were removed and 0.1 mL DMSO was added and incubated for 15 min to solubilize the MTT. Optical density was measured at 570 nm with an iMark microplate reader (Bio-Rad, Hercules, CA, USA). Cell viability was expressed as a percentage of control cells.

### 4.7. Stimulation of Cells with LPS or LPS Plus IFN-γ

For iNOS, NO, COX-2, and cytokine analysis, mouse peritoneal macrophages were stimulated with 100 ng/mL LPS (Sigma, St. Louis, MO, USA) plus 0.5 ng/mL IFN-γ (BD Pharmingen, San Diego, CA, USA) or LPS alone for 24 h, and samples were added simultaneously. For signaling molecule analysis, mouse peritoneal macrophages were pretreated with samples for 1 h and then stimulated with LPS for 15 min or LPS plus IFN-γ for 2 h.

### 4.8. Determination of Nitrite Accumulation

Supernatant from the above culture was incubated with an equal volume of Griess reagent (Sigma, St. Louis, MO, USA) for 15 min at room temperature. Optical density was measured at 540 nm with the microplate reader.

### 4.9. Cytokine Analysis

Levels of TNF-α and IL-6 were determined by enzyme-linked immunosorbent assay according to the manufacturer’s protocol (BD Pharmingen, San Diego, CA, USA).

### 4.10. Western Blot Analysis

Cells were rinsed in cold PBS and then lysed on ice in 0.1 mL of RIPA buffer (50 mM Tris-HCl, pH 7.5; 150 mM NaCl; 1 mM EDTA; 20 mM NaF; 0.5% NP-40; and 1% Triton X-100) containing a phosphatase inhibitor cocktail (Sigma, St. Louis, MO, USA) and a protease inhibitor cocktail (Sigma, St. Louis, MO, USA). After centrifugation at 13,000× *g* for 10 min, supernatants were collected. Protein concentrations were determined using the Bradford protein assay reagent (Bio-Rad, Hercules, CA, USA), and the samples were diluted with sodium dodecyl sulfate (SDS) buffer and boiled for 3 min. The samples were separated on an 8% or 10% SDS-polyacrylamide gel and were transferred to polyvinylidene fluoride membranes. The membranes were then blocked with 5% skim milk in Tris-buffered saline with 0.1% Tween 20 (TBST) for 1 h. The membranes were incubated with iNOS, IκBα, GAPDH, or tubulin (Santa Cruz Biotechnology, Santa Cruz, CA, USA), COX-2 (Cayman, Ann Arbor, MI, USA), phospho-JNK (T183/Y185), JNK, phospho-ERK1/2 (T202/T204), ERK, phospho-p38 (T180/Y204), p38, phospho-TBK1(S172), TBK1, phospho-STAT1 (Y701), and STAT1 (Cell Signaling Technology, Beverly, MA, USA) diluted in 5% skim milk in TBST overnight at 4 °C. The blots were washed with TBST and incubated for 1 h with anti-rabbit horseradish peroxidase-conjugated antibodies. Immunoreactive bands were detected with EzWestLumi plus (ATTO, Tokyo, Japan) and analyzed using an EZ-Capture MG (ATTO, Tokyo, Japan).

### 4.11. Luciferase Assay

NF-κB and AP-1 transcriptional activity was measured using RAW264.7 cells transfected with the pGL4.32 containing five copies of an NF-κB response element or pGL4.44 containing six copies of an AP-1 responsive element and the firefly luciferase reporter gene (luc2P) (Promega, Madison, WI, USA). The transfected RAW264.7 cells were plated to 96 well plates overnight. After a media change, cells were pretreated with robinin for 2 h and then stimulated with LPS for 6 h. Luciferase activity was measured using the Dual-Glo^®^ luciferase assay system (Promega, Madison, WI, USA).

### 4.12. Statistical Analysis

Data were analyzed by student *t* test or ANOVA followed by the Tukey test using IBM SPSS 22 software. *p* Values less than 0.05 were considered significant.

## Figures and Tables

**Table 1:** Contents of major flavonoids in kudzu leaf and root. All units: mg/g dry weight.

	Robinin	Puerarin	Yield of Crude Extract
Leaf	4.61 ± 0.18	N.D.	110.2
Root	N.D.	43.54 ± 0.83	253.4

N.D. “not detected”.

## Supplementary Materials

Supplementary materials can be found at http://www.mdpi.com/1422-0067/19/5/ 1536/s1.
