# Supplementary material for: Kudzu Leaf Extract Suppresses the Production of Inducible Nitric Oxide Synthase, Cyclooxygenase-2, Tumor Necrosis Factor-Alpha, and Interleukin-6 via Inhibition of JNK, TBK1 and STAT1 in Inflammatory Macrophages
Source: Int J Mol Sci. 2018 May 22;19(5):1536. doi: 10.3390/ijms19051536 (PMC5983698; doi:10.3390/ijms19051536)
Supplement: Supplementary file 1 [file ijms-19-01536-s001.pdf]

Supplementary data

Effects of kudzu leaf extract and robinin on NF-kB transcriptional activity

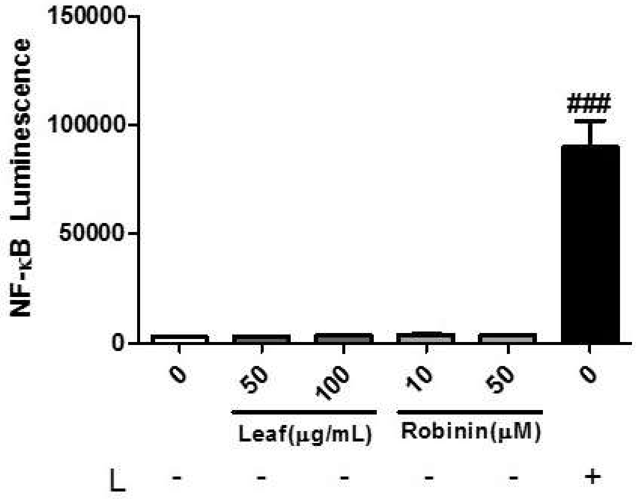

Figure S1. Effects of kudzu leaf extract and robinin on NF-kB transcriptional activity. RAW264.7 cells were transfected with an NF-κB-dependent reporter gene. Cells were pretreated with kudzu leaf extract or robinin for 2 h and then stimulated with LPS for 6 h. Luciferase activity was measured using the Dual-Glo® luciferase assay system (n=3). ### *P* <0.005 vs. control (-L).

Identification of an isolated compound by ^{1}H-NMR and ^{13}C-NMR

^{1}H and ^{13}C NMRs (JNM-LA 400, FT-NMR, JEOL, Japan) were conducted using a deuterated methanol solvent (Sigma-aldrich Co., USA) for identifying the structure of the isolated compound. Five milligrams of the isolated compound was dissolved into 1 mL of a deuterated methanol and run NMR processes.

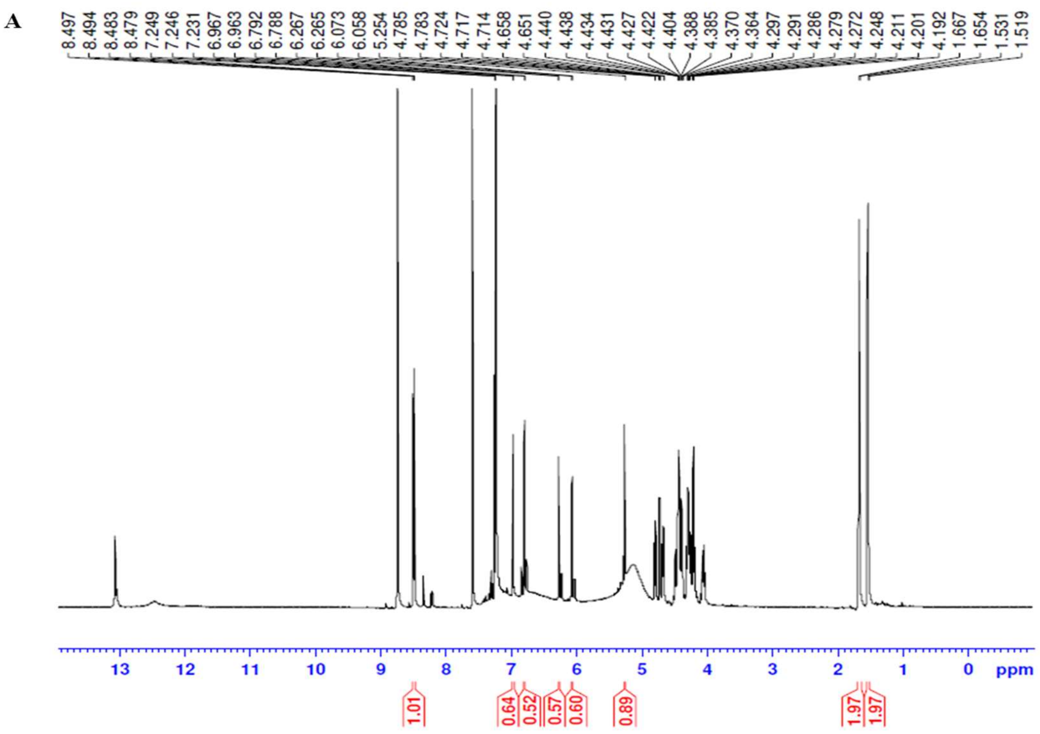

Figure S2. ^{1}H-NMR and ^{13}C-NMR chromatograms of robinin. A: ^{1}H-NMR, B: ^{13}C-NMR

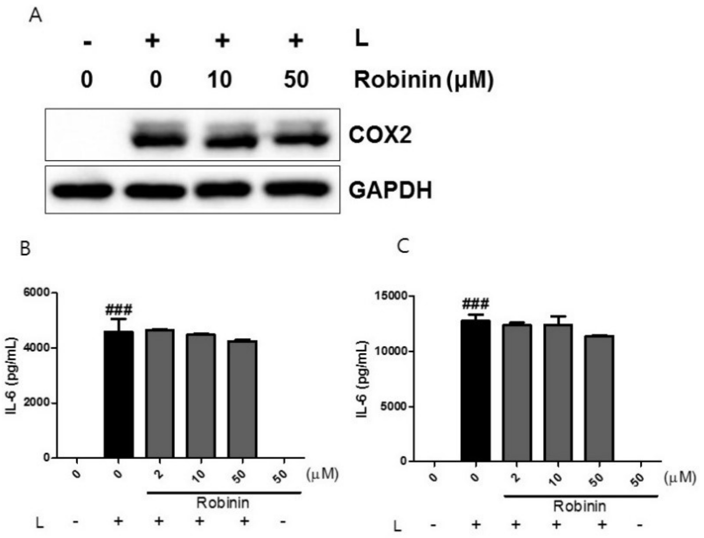

Figure S3. Effect of robinin on COX-2 and IL-6 in mouse peritoneal macrophages. A: Cells were stimulated with LPS (L) in the presence of robinin for 24 h. The level of COX-2 protein was analyzed by Western blotting using GAPDH as an internal control. One of three independent experiments is shown. B-C: The levels of IL-6 at 6 h (B) and 24 h (C) in the supernatant was measured by ELISA (n=3). ### *P* <0.005 vs. control (-L).
